# TRPs in Pain Sensation

**DOI:** 10.3389/fphys.2017.00392

**Published:** 2017-06-09

**Authors:** Isaac Jardín, José J. López, Raquel Diez, José Sánchez-Collado, Carlos Cantonero, Letizia Albarrán, Geoffrey E. Woodard, Pedro C. Redondo, Ginés M. Salido, Tarik Smani, Juan A. Rosado

**Affiliations:** ^1^Cell Physiology Research Group, Department of Physiology, University of ExtremaduraCáceres, Spain; ^2^Department of Surgery, Uniformed Services University of the Health SciencesBethesda, MD, United States; ^3^Department of Medical Physiology and Biophysics, Institute of Biomedicine of Sevilla, University of SevilleSevilla, Spain

**Keywords:** calcium entry, TRPs, TRPA1, TRPV1, noxious sensation

## Abstract

According to the International Association for the Study of Pain (IASP) pain is characterized as an “unpleasant sensory and emotional experience associated with actual or potential tissue damage”. The TRP super-family, compressing up to 28 isoforms in mammals, mediates a myriad of physiological and pathophysiological processes, pain among them. TRP channel might be constituted by similar or different TRP subunits, which will result in the formation of homomeric or heteromeric channels with distinct properties and functions. In this review we will discuss about the function of TRPs in pain, focusing on TRP channles that participate in the transduction of noxious sensation, especially TRPV1 and TRPA1, their expression in nociceptors and their sensitivity to a large number of physical and chemical stimuli.

## Introduction

Cytosolic free Ca^2+^ concentration ([Ca^2+^]_c_) is a key factor for the regulation of a large variety of cellular functions, ranging from short-term processes, such as muscle contraction, exocytosis, or platelet aggregation, to long-term events, including cell proliferation or apoptosis (Berridge et al., 2000). Physiological agonists modulate [Ca^2+^]_c_ through the regulation of a number of Ca^2+^ transport mechanisms, based on the activation of more or less Ca^2+^ selective channels and transporters. Agonist-induced Ca^2+^ mobilization consist, among others, of (1) the release of Ca^2+^ from agonist-sensitive Ca^2+^ stores, mostly the endoplasmic reticulum (ER) and acidic organelles (Lopez et al., 2005; Galione, 2006; Aulestia et al., 2011), (2) extracellular Ca^2+^ entry through plasma membrane permeable channels (Salido et al., 2009a), (3) cytosolic Ca^2+^ clearance either by Ca^2+^ uptake into intracellular stores (Lipskaia et al., 2014) or Ca^2+^ extrusion across the plasma membrane (Redondo et al., 2005), and (4) Ca^2+^ buffering with the participation of the mitochondria (Montero et al., 2001). While Ca^2+^ release from the finite intracellular Ca^2+^ compartments has been reported to regulate different cellular events, Ca^2+^ entry from the extracellular medium is required for the replenishment of the internal stores and also for full activation of different cellular functions. Ca^2+^ entry might occur through a variety of mechanisms, which might be grouped in voltage-operated and receptor-operated Ca^2+^ influx processes. In turn, according to the activation route, receptor-operated Ca^2+^ entry might be classified into receptor-mediated, second messenger-operated and store-operated Ca^2+^ entry mechanisms. The simplest mechanism is receptor-mediated Ca^2+^ influx, which occurs through channels allosterically regulated by agonist binding. Second messenger-operated Ca^2+^ entry requires the generation of a second messenger that directly gates the channel. On the other hand, store-operated Ca^2+^ entry (SOCE) is regulated by the filling state of the intracellular Ca^2+^ stores, mainly the ER (Putney, 1986), but also acidic organelles (Zbidi et al., 2011). According to this, a reduction in the intraluminal Ca^2+^ concentration results in the opening of channels in the plasma membrane (Putney, 1986).

Among the wide variety of Ca^2+^-permeable channels identified, TRP channels play a relevant functional role in mammalian cells. TRP channels were identified in a *Drosophila* mutant with visual defects, where a mutation in a channel permeable to Na^+^ and Ca^2+^ leads to transient, rather than sustained, receptor potential in the photoreceptors (Minke, 1977). Since the identification of the mammalian homologs of the *Drosophila* TRPs in 1995 (Wes et al., 1995; Zhu et al., 1995) these channels have been proposed as candidates to conduct both second messenger- as well as store-operated Ca^2+^ entry.

TRP channels are a group of ion channels located in the plasma membrane as well as in the membrane of a number of intracellular organelles, where they participate in the homeostasis of intracellular Ca^2+^, as well as other ions, such as Mg^2+^ (Fleig and Penner, 2004; Ambudkar et al., 2007; Salido et al., 2009b). Since TRP proteins were first described a number of isoforms have been identified, which are grouped into seven subfamilies: TRPC, TRPV, TRPM, TRPP, TRPML, TRPA, and TRPN (the latter only expressed in fish, flies, and worms) and each subfamily includes one or more members (Montell et al., 2002; Li et al., 2011).

The structure of TRP channels comprises six membrane-spanning helices with a pore-forming loop between the last two transmembrane segments. The N- and C-terminal segments are located in the cytosol and vary in the number of amino acids and the functional motifs among the different subfamilies. Thus, the N termini of TRPC, TRPA, TRPV, and TRPN subfamilies contain between 4 and 30 tandem copies of ankyrin repeat domains, involved in protein-protein interaction (Latorre et al., 2009). Furthermore, the cytoplasmic N and/or C-termini of TRPC, TRPM, TRPP, and TRPV channels have been reported to contain coiled coil domains, which play an important role in the assembly of homomeric and heteromeric complexes (Lepage and Boulay, 2007; Schindl and Romanin, 2007) as well as in the interaction with the ER Ca^2+^ sensor STIM1 (Lee et al., 2014). TRPC, TRPV, and TRPM subfamilies also contain a conserved TRP box, a short hydrophobic region located just C-terminal of the putative last transmembrane segment (Nilius et al., 2006). Certain TRP members are regulated by cytosolic Ca^2+^ through the interaction with C-terminal located EF-hand motifs, including TRPA1, TRPML1, and TRPP2 (Tsiokas, 2009), or calmodulin and IP3 receptor-binding regions, this is the case of TRPC, TRPM, and TRPV members (Tang et al., 2001; Dionisio et al., 2011). Finally, other more restricted motifs have been reported in different TRP members, including the tubulin-binding domain reported in TRPV1 (Sardar et al., 2012), the kinase domain of TRPM6 and TRPM7 (Schlingmann and Gudermann, 2005), the conserved proline-rich region, downstream of the EWKFAR motif, responsible for the interaction with Homer proteins and immunophilins (Yuan et al., 2003; Sinkins et al., 2004; Jardin et al., 2013; Lopez et al., 2013; Dionisio et al., 2015) or the voltage sensing domain reported in TRPV1, TRPV3, TRPM8, and TRPM4 (Nilius et al., 2003, 2005), among others.

TRP channels are activated and modulated by a wide variety of chemical and physical stimuli including receptor occupation via activation of phospholipase C, which, in turn, leads to the hydrolysis of phosphatidylinositol 4,5-bisphospate (PIP_2_) and the generation of lipid messengers, biosynthesis of IP_3_, and subsequent Ca^2+^ release from the intracellular stores, the activation of serine/threonine or tyrosine kinases or ligand binding, including exogenous ligands, such as capsaicin or allyl isothiocyanate, and endogenous molecules, including eicosanoids, diacylglycerol, phosphoinositides, purine nucleotides, or inorganic ions, such as Ca^2+^ and Mg^2+^ (Harteneck et al., 2011; Vetter and Lewis, 2011).

The sensitivity of TRP channels to a number of physical and chemical stimuli allows these channels to be essential components of different sensory processes, such as vision, hearing, taste, tactile and thermal sensation, redox status, or pain (Voets et al., 2005; Woodard et al., 2007; Wetsel, 2011; Feng, 2014; Ogawa et al., 2016).

## TRP channels and nociception

### Pain and nociception

Pain is a subjective unpleasant sensory experience that might be associated to real or potential damage. Noxious stimuli are detected by pain receptors or nociceptors, nerve endings that specifically respond to damaging stimuli and transmit the information to the spinal cord, through which the message is transmitted to higher nerve centers, including the brain stem reticular formation, thalamus, somatosensory cortex, and limbic system (Osterweis et al., 1987). Nociception, therefore, is the process of transmission of noxious signals by nociceptors in the primary afferent nerve fibers (Dai, 2016). Noxious stimuli are classified into chemical, mechanical and thermal. The transduction of nociception includes several chemical compounds that might be released by damaged tissue, such as K^+^, histamine and serotonin, or generated by enzymes activated by tissue damage, including prostaglandins, leukotrienes, or bradikinin (Schaible et al., 2011; Viguier et al., 2013).

A major function of the nociceptors is to detect potentially damaging stimuli with a threshold that allows perform activities without pain but sensitive enough to warn of the risk of damage (Patapoutian et al., 2009). The detection of noxious stimuli by nociceptors involves the expression of nociceptive ion channels, which basically define the functional properties of nociceptors. The largest group of nociceptive ion channels is the TRP channel family (Clapham, 2003; Patapoutian et al., 2009), especially TRPV1 and TRPA1 members. Activation of nociceptive TRP ion channels in sensitive (i.e., dorsal root ganglion, DRG) neurons leads to the influx of Na^+^ and Ca^2+^ across the plasma membrane resulting in membrane depolarization that, in turn, might trigger voltage-gated ion channel-dependent action potentials (Gees et al., 2010) that transmit the information to the spinal cord and the higher nerve centers as described above.

### Nociceptive TRP ion channels

#### TRPV1

**Figure 1:**
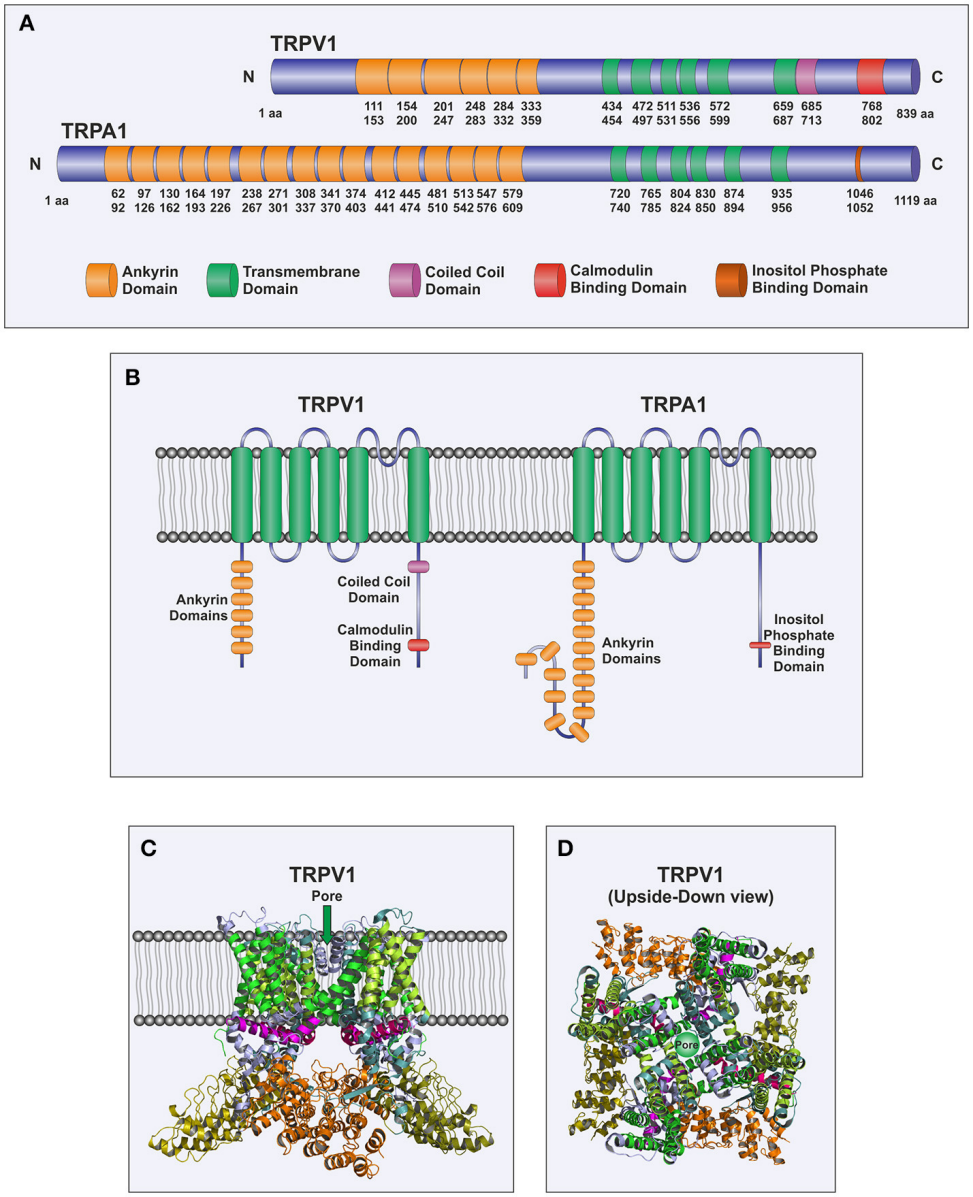
Molecular structure of TRPA1 and TRPV1. **(A)** Scheme of TRPV1 and TRPA1 channels depicting individual domains. Numbers correspond to amino acids positions of human TRPV1 and TRPA1, respectively. **(B)** Cartoon representing TRPV1 and TRPA1 monomers morphology within a bilayer membrane. The channel spans the membrane up to six times with the pore located between transmembrane domains (TM) 5 and 6, and both N-terminal and C-terminal domains situated in the cytosol. **(C,D)** Cartoons depicting the tetrameric assembly of TRPV1 subunits based on the X-ray crystal structure of *Rattus norvegicus* as described in Cao et al. (2013). As mentioned above, the ion permeation pathway is formed by TM5 and TM6, while the remaining TM domains 1–4, surround the pore. **(C)** Represents a frontal view of the channel while **(D)** sketches an upside-down perspective.

TRPV1 is one of the six members of the TRPV subfamily and is involved in the detection of noxious sensation (Caterina and Julius, 2001). TRPV1 has been found to be highly expressed in the plasma membrane of nociceptive DRG neurons (Caterina and Julius, 2001). Furthermore, functional expression of this channel has also been reported to be expressed in the ER of DRG neurons, where it is involved in Ca^2+^ efflux from the ER upon stimulation with vanilloids; although its sensitivity to agonists is smaller when located in the ER membrane probably due to a mechanism mediated by calmodulin, which might be important for neuronal biology (Gallego-Sandin et al., 2009). The structure of TRPV1 follows the pattern of the TRP channels, with six transmembrane spanning domains, six ankyrin repeats in the N-terminus and a large C-terminal region (Cao et al., 2013; Figure 1). Three splice variants of TRPV1 have been described: VR.5'sv, TRPV1b, and TRPV1var. VR.5'sv (vanilloid receptor 5' splice variant) shows a shorter N-terminal region due to both an alternative initiation of translation and the lack of transcription of an exon resulting in loss of 60 amino acids in the N-terminus (Schumacher et al., 2000) and do not respond to capsaicin (Eilers et al., 2007). TRPV1b shows a modification in the N-terminal region encoded by exon 7 that leads to loss of 10 amino acids (Wang et al., 2004) and, as well as the VR.5'sv variant, has been propose to function as a dominant-negative channel subunit (Pecze et al., 2008; Schumacher and Eilers, 2010). TRPV1var is generated by a failure to splice out intron 5, thus leading to translation of a portion of the N-terminal region that lacks the transmembrane spanning domains and the C-terminal intracellular region (Tian et al., 2006). It has been reported that TRPV1var, when coexpressed with the full-length TRPV1 subunits, might modulate its responses, for instance, it has been shown to increase the response of TRPV1 to resiniferatoxin (Tian et al., 2006).

TRPV1 is a polymodal channel sensitive to different physical and chemical stimuli, including heat (see above), pH under 5.9 (Tominaga et al., 1998), and mechanical stimuli (Walker et al., 2003). In addition, TRPV1 is activated by a variety of ligands (Table 1) including vanilloids, such as capsaicinoids (the most representative is capsaicin, the major pungent constituent of *Capsicum* fruit; Caterina et al., 1997) and resiniferanoids (Szallasi and Blumberg, 1989), α, β-unsaturated dialdehydes isolated from a variety of plants, fungi, algae, sponges, arthropods, and molluscs (Jonassohn et al., 1995), cannabinoids from *Cannabis sativa* (Bisogno et al., 2001), ginsenosides found in the ginseng *Panax ginseng* (Jung et al., 2001), a number of animal-derived toxins, such as VaTx1, VaTx2, and VaTx3 found in the venom of the tarantula *Psalmopoeus cambridgei* activates TRPV1 channels (Siemens et al., 2006) while other toxins, including agatoxin 489 and agatoxin 505, from the spider *Agelenopsis aperta*, and the analgesic polypeptide HC1, from the see anemona *Heteractis crispa*, elicits TRPV1 inhibition (Kitaguchi and Swartz, 2005; Andreev et al., 2008). Furthermore, a number of endogenous molecules, known as endovanilloids, including leukotriene B4 and 12-S-HPETE and anandamide (a cannabinoid receptor agonist) have been found to be potent activators of TRPV1 channels (Di Marzo et al., 2002; Hermann et al., 2003). On the other hand, alkaloids, such as nicotine (from *Nicotiana tabacum*) or yohimbine (from the bark of the tree *Pausinystalia yohimbe*), phenols like grifolin, neogrifolin, and albaconol (present in the mushroom *Albatrellus confluens*) or acetylsalicylic acid have been found to inhibit the activity of TRPV1 channels, for a review see Vetter and Lewis (2011). Endogenous modulators of TRPV1 activity include noradrenaline, which is able to attenuate capsaicin-activated response by ~60%, a mechanism mediated by activation of α2 adrenergic receptors that has been reported to underlie the inhibition of the incoming noxious stimuli at the dorsal horn of the spinal cord (Chakraborty et al., 2017).

**Table 1:** Agonists and antagonists of TRPV1 and TRPA1 channels.

**Channel**	**Agonist**	**Potency (EC_50_)**	**Antagonist**	**Potency (IC_50_)**	**References**
TRPV1	Capsaicin	0.04–1 μM	Agatoxin 489	0.3 μM	Jung et al., 2001; Behrendt et al., 2004; Varga et al., 2005; Rami et al., 2006; Harteneck et al., 2011; Planells-Cases et al., 2011; Vetter and Lewis, 2011; Xia et al., 2011
	Eugenol	1 mM	Agatoxin 505	0.3 μM	
	Resiniferatoxin	39 nM	APHC1	54 nM	
	Polygodial	5 μM	Capsazepine	420 nM	
	Cinnamodial	0.6 μM	Nicotine	1 mM	
	Isovelleral	100 nM	Yohimbine	25 μM	
	Cannabidiol	3 μM	Acetylsalicylic acid	1 μM	
	Ginsenoside Rc	?	Grifolin	26 μM	
	VaTx1	12 μM	Neogrifolin	7 μM	
	VaTx2	3 μM	Albaconol	17 μM	
	VaTx3	0.3 μM	BCTC	35 nM	
	Leukotriene B4	30 μM	AMG-517	32 nM	
	12-S-HPETE	10 μM	SB366791	651 nM	
	Anandamide	30 μM			
TRPA1	Allyl isothiocyanate	1–6.5 μM	Camphor	0.6 mM	Karashima et al., 2007; Trevisani et al., 2007; Cruz-Orengo et al., 2008; Eid et al., 2008; Sculptoreanu et al., 2010; Harteneck et al., 2011; Vetter and Lewis, 2011; Trevisan et al., 2014; Wei et al., 2009; McGaraughty et al., 2010; Brenneis et al., 2011; Sisignano et al., 2012
	Cinnamaldehide	60μM	HC-030031	6.2 μM	
	Methyl salicylate	600 μM	A-967079	67 nM	
	Allicin	7.5 μM	Chembridge-5861528	?	
	Ajoene	0.5 μM			
	Diallyl trisufphide	0.5 μM			
	Hydroxy-α-sanshool	69 μM			
	Acrolein	5 μM			
	Crotonaldehyde	16 μM			
	Δ9 tetra-hydrocannabinol	12 μM			
	Cannabinol	20 μM			
	Hydrogen peroxide	?			
	Nitrooleic acid	?			
	4-hydroxy-2-nonenal	27 μM			
	15-deoxy- Δ12,14-PGJ2	?			
	5,6-EET	?			
	8,9-EET	?			
		?			

#### TRPA1

TRPA1, also known as P120 and ANKTM1, is the sole member of the TRPA subfamily. It was first described in human fibroblasts where its expression is lost after oncogenic transformation (Jaquemar et al., 1999). TRPA1 has been found to be expressed in peptidergic nociceptors, as well as in a number of non-neuronal cells, including keratinocytes (Atoyan et al., 2009), megakaryocytes (Albarran et al., 2013) or enterochromaffin cells (Nozawa et al., 2009), and tissues (for a review see Benemei et al., 2014).

In addition to TRPV1, TRPA1 is specialized in the transduction of noxious stimuli in mammals. In fact, a certain degree of interaction between both channels has been reported. TRPV1 is expressed in most TRPA1-expressing neurons and about 30% of TRPV1-expressing sensory neurons also exhibit TRPA1 expression (Story et al., 2003). Furthermore, TRPV1 has been reported to influence several features of the TRPA1 channel, such as voltage–current relationships and open probability (Staruschenko et al., 2010). Further pieces of evidence for the functional interaction between both channels comes from studies reporting that the biophysical properties of TRPA1 are different when TRPA1 is expressed alone or coexpressed with TRPV1 and that the TRPV1 and TRPA1 agonists, capsaicin and mustard oil, are able to induce heterologously desensitization of TRPA1 and TRPV1 via calcineurin-dependent and independent pathways, respectively (Ruparel et al., 2008).

The structure of TRPA1 shows the features of the TRP family and consists of six membrane-spanning domains and a presumed pore-forming region between the fifth and sixth transmembrane domains. Its N- and C-terminal segments are predicted to be located in the cytoplasm (Figure 1). In addition, an unusual and characteristic feature of TRPA1 is the presence of a very long N-terminus, which contains at least 16 predicted ankyrin repeat domains (Story et al., 2003; Paulsen et al., 2015). It is the only mammalian TRP channel with such high number of ankyrin repeats, which might provide the protein a certain degree of elasticity, as well as, the ability to interact with other proteins, especially those of the cytoskeleton (Corey et al., 2004; Sotomayor et al., 2005).

TRPA1 is a polymodal ion channel that can be activated by a number of physical and chemical stimuli. Among the physical stimuli, TRPA1 is sensitive to temperature. The 10 thermo-TRP channels identified to date, including TRPV1-4, TRPM2, TRPM4, TRPM5, TRPM8, TRPC5, and TRPA1, are activated by different temperature ranges. The mammalian TRPs activated by heat are TRPV2 (activated at temperatures over 52°C), TRPV1 (sensitive to temperatures over 42°C), TRPV4 (activated by temperatures between 27 and 42°C), TRPV3 (by temperature over 33°C), TRPM2 (sensitive to temperatures between 35 and 42°C), TRPM4 and TRPM5 (sensitive to temperatures between 15 and 35°C). On the other hand, TRPC5 activity is potentiated at temperatures below 30°C, TRPM8 is sensitive to temperatures below 25°C and TRPA1 is activated at temperatures below 17°C (Caterina et al., 1999; Dhaka et al., 2006; Vriens et al., 2014). As TRPV1 has been associated to painful heat, TRPA1 has been reported to be associated to noxious cold sensation (Patapoutian et al., 2003). The thermal sensitivity of TRPA1 is conserved throughout evolution, although the range of temperatures that activates the channel differs among the distinct vertebrates, thus, in reptiles and amphibians TRPA1 is sensitive to heat and, in certain snakes, TRPA1 provides sufficient thermal sensitivity for infrared detection (Poletini et al., 2015; Kang, 2016).

In addition, TRPA1 can be activated by a number of chemical stimuli (Table 1), including exogenous compounds, such as isothiocyanates, cinnamaldehyde, and methyl salicylate (the pungent compounds associated to burning sensation present in mustard oil, wasabi, horseradish, cinnamon and wintergreen oil; Bandell et al., 2004), allicin, ajoene, and diallyl sulfides (organosulfur compounds present in garlic; Bautista et al., 2006), acrolein, and crotonaldehyde (present in cigarette smoke; Andre et al., 2008), cannabinoids, such as Δ^9^ tetra-hydrocannabinoland cannabinol (Jordt et al., 2004), alkylamides, including hydroxy-α-sanshool (one of the compounds of the Szechuan pepper; Riera et al., 2009; Vetter and Lewis, 2011), or endogenous compounds such as hydrogen peroxide (Trevisan et al., 2014), nitro-oleic acid, a byproduct of nitric oxide (Sculptoreanu et al., 2010), 4-hydroxy-2-nonenal (Trevisani et al., 2007), the cyclopentenone prostaglandin D2 metabolite 15-deoxy- Δ^12, 14^-prostaglandin J_2_ (Cruz-Orengo et al., 2008), and different epoxyeicosatrienoic acids (EET), including 5,6-EET (Sisignano et al., 2012) and 8,9-EET (Brenneis et al., 2011). By contrast, a number of TRPA1 antagonists have been identified, including the synthetic HC-030031, its derivative chembridge-5861528 or A-967079, among others (Table 1). Most exogenous compounds activate TRPA1 channels by covalent modification of cysteines and lysines in the N-terminus (Hinman et al., 2006; Macpherson et al., 2007; Nilius et al., 2011), although it remains to be determined the mechanism of activation of the channel by certain endogenous compounds.

In addition to TRPV1 and TRPA1, other TRP members have been associated to noxious sensation, including TRPM3, expressed in a number of small-diameter sensory neurons from dorsal root and trigeminal ganglia where it is involved in the nocifensive response to heat (Vriens et al., 2011), TRPV4, presented as an osmo-transducer in primary afferent nociceptive nerve fibers (Alessandri-Haber et al., 2003), TRPC1 and TRPC6, which cooperate with TRPV4 in the mediation of hyperalgesia to mechanical and hypotonic stimuli induced by inflammatory mediators (Alessandri-Haber et al., 2009), TRPV3, a channel sensitive to farnesyl pyrophosphate that is involved in the sensitivity to noxious heat (Bang et al., 2010) TRPM8, involved in cold hyperalgesia and tactile allodynia (Salat and Filipek, 2015), TRPC3, associated to the mediation of store- and receptor-operated Ca^2+^ entry in DRG neurons (Alkhani et al., 2014), TRPC4, which is required for the detection or transmission of colonic visceral pain sensation, and TRPC5, which, together with TRPC4, is relevant for pain hypersensitivity and neuropathic pain (Westlund et al., 2014; Wei et al., 2015); however, the involvement of these channels in pain detection or transmission has been less characterized than that of TRPV1 or TRPA1.

## Nociceptive TRP channels and pain pathologies

Nociceptive TRP channels have been found to be involved in a number of pain modalities, including inflammatory pain, neuropathic pain, visceral pain, and pain associated to certain pathological conditions, including cancer or migraine (Mickle et al., 2016).

The involvement of TRPV1 in inflammatory pain is the most prominent among the TRP channels. TRPV1 antagonists have been shown to be efficient attenuating thermal hyperalgesia induced under inflammatory conditions and increasing the noxious heat threshold (Tekus et al., 2010) and similar results have been obtained in TRPV1 lacking mice models (Davis et al., 2000). Further evidence supporting the role of TRPV1 in inflammatory pain comes from studies reporting that TRPV1 is essential for the analgesia induced by electroacupuncture in a mouse model of inflammatory pain (Liao et al., 2017). In addition to TRPV1, TRPA1 has been presented as a candidate to mediate inflammatory mechanical hyperalgesia as well as cold hyperalgesia under inflammatory conditions (Eid et al., 2008; Da Costa et al., 2010). Furthermore, TRPA1 has been reported to modulate inflammation and pruritogen responses in allergic contact dermatitis. TRPA1 is involved in skin edema, leukocyte infiltration and antihistamine-resistant scratching in mice treated with oxazolone (Liu et al., 2013).

Neuropathic pain occurs as a result of nerve injury. The role of TRPV1 has been demonstrated in neuropathic pain associated to diabetes or the administration of chemotherapeutics (Bourinet et al., 2014). A more recent study has revealed a high coexpression between TRPV1 and different sensitizing agents, such as PKCε, during the development of neuropathic pain (Malek et al., 2015) and blockade of this mechanism by quercetin has been found to attenuate paclitaxel-induced neuropathic pain (Gao et al., 2016). TRPA1 has also been proposed to mediate mechanical hyperalgesia and allodynia during neuropathic pain in diabetic patients or derived from the administration of chemotherapeutics, probably mediated by the synthesis of reactive oxygen and nitrogen species (Kim and Hwang, 2013; Huang et al., 2017), which are well-known TRPA1 activators (Trevisan et al., 2016). TRPA1 and TRPV1 have also been involved in the development of migraine, which can be activated by a number of TRPA1 agonists (Benemei et al., 2014) and might be attenuated by repeated desensitizing administration of capsaicin to the nasal mucosa (Fusco et al., 2003). Furthermore, ethanol, a well-known trigger of migraine, has been reported to induce TRPV1 activation (Nicoletti et al., 2008). Activation of TRP channels, such as TRPV1 and TRPA1, has been reported to induce the trigeminal calcitonin gene-related peptide pathway, which mediate neurogenic inflammation, thus leading to the migraine attacks (Benemei et al., 2013).

Visceral pain occurs in internal organs and its transduction involves different TRP family members, including TRPV1, TRPA1, and TRPM8. Silencing the expression of TRPV1 by RNAi has been reported to attenuate visceral pain *in vivo* (Christoph et al., 2006). Consistent with this, a more recent study has revealed that decreased expression of miR-199 in irritable bowel syndrome, which results in enhanced expression of TRPV1, leads to increased visceral hypersensitivity (Zhou et al., 2016). On the other hand, the luminal gasotransmitter hydrogen disulphide has been reported to induce colonic pain and hyperalgesia via activation of Ca_v_3.2 and TRPA1 channels (Tsubota-Matsunami et al., 2012). Furthermore, the TRPA1 agonist ASP7663 has been reported to prevent constipation (a gastrointestinal motility disorder) when administered orally, and induce analgesic abdominal effects when it is intravenously administered (Kojima et al., 2014). Finally, TRPM8 has been found to play a relevant role in overactive bladder and painful bladder syndrome and it has been reported that administration of the TRPM8 channel blocker AMTB is able attenuate this syndrome in rats (Lashinger et al., 2008).

Chronic pain is also a multidimensional complication of cancer or its treatment. The role of TRPV1 in bone cancer pain has been widely investigated. TRPV1 has been found to be associated to bone cancer pain, as demonstrated by pharmacological inactivation of TRPV1 as well as disruption of the TRPV1 gene (Ghilardi et al., 2005). Furthermore, the TRPV1 antagonist SB366791 has been reported to potentiate the analgesic effect of intraperitoneal administration of morphine in a mouse model of bone cancer pain. The expression of TRPV1, as well as the TRPV1-dependent currents, have been found to be enhanced upon the development of bone cancer in DRG neurons. In these cells, capsaicin-mediated currents were potentiated by administration of lysophosphatidic acid through a mechanism dependent on PKCε but independent on PKA and the small GTPase Rho (Pan et al., 2010). Two more recent studes have revealed that the up-regulated expression and function of TRPV1 in bone cancer pain might be attributed to the the presence of tumor tissue-derived endogenous formaldehyde, which enhances TRPV1 expression via mitogen-activated protein kinase and PI3K, but independently on PKC (Han et al., 2012), as well as the regulatory effects of insulin-like growth factor-1 (Li et al., 2014). Finally, JAK/PI3K-dependent TRPV1 up-regulation has been reported to be involved in peripheral sensitization and bone cancer-induced pain evoked by interleukin-6 (Fang et al., 2015). TRPV1 and TRPA1 have also been found to be involved in neuropathic pain due to the administration of chemotherapeutics, including oxaliplatin (Park et al., 2015), 5-fluorouracil (Yamaguchi et al., 2016), or docetaxel (Huang et al., 2017).

Currently, there is a body of studies and clinical trials identifying new antagonists of the nociceptive TRPs and characterizing their effects in the *in situ* attenuation of pain transduction at the nociceptors.

## Author contributions

JR, IJ, and JL drafted the manuscript. IJ performed the figure. RD, JS, CC, LA, and TS performed the bibliographic revision. GW, GS, and PR revised the manuscript and performed the final check.

### Conflict of interest statement

The authors declare that the research was conducted in the absence of any commercial or financial relationships that could be construed as a potential conflict of interest.
